# Hemiplegia from Tuberculous Meningitis

**Published:** 1908-05-02

**Authors:** 


### Illustrative Cases.

### HEMIPLEGIA FROM TUBERCULOUS MENINGITIS.

The following case is illustrative of two chief points, namely—

(1) That tuberculous meningitis may give rise to hemiplegia, and thus simulate a unilateral brain lesion.

(2) That ophthalmoscopic examination in such cases may sometimes be extremely valuable in assisting the diagnosis by discovering a choroidal tubercle.

The patient was an infant, aged a year and two months, born of healthy parents, and breast-fed until it was seven months old. It was then weaned, and given for the most part cow's milk with a little barley water, some of the feeds being thickened with a little prepared food. It had seemed to be in perfectly good health until, without apparent cause, and quite apart from any injury, it had a severe attack of repeated vomiting, accompanied by extreme restlessness and much crying. This attack passed off, though the infant was persistently more fretful than it had been previously; but at the end of ten days it almost suddenly developed hemiplegia. There was no unconsciousness. No convulsions occurred. The ears were perfectly natural, and there had at no time been any discharge from either. The infant no longer screamed or cried, but lay in a dull torpid state, with paresis and some rigidity of the left arm and left leg. There was no obvious facial paralysis. The pupils were widely dilated, but they reacted to light.

Ophthalmoscopic examination showed no optic neuritis, but there was a very distinct choroidal tubercle in the left eye. This by itself clinched the diagnosis of general tuberculosis, although the child's temperature was normal or subnormal, and there were no abnormal physical signs in the chest or abdomen. Kernig's sign was absent, and tâche cérébrale was no more marked than it is in many children suffering from other forms of ill health.

It is interesting to note that there was at this time no retraction of the head, and there had been no convulsions nor strabismus. Indeed, convulsions were entirely absent throughout. Retraction of the head first developed on the twenty-second day, but it never became extreme.

There was no pyrexia at all until the fortieth day of the illness, and then it rose to 103°F., synchronously with the appearance of bilateral otorrhœa. It is probable that pyrexia is seldom due to the tubercles in the meninges in these cases, but rather to the extent of the tubercles in the other parts of the body. If a case of tuberculous meningitis exhibits considerable pyrexia, apart from a terminal hyperpyrexia, it is probable that tubercles are very numerous in other viscera besides the brain and its meninges; and that when the chief site of the trouble is meningeal, with a minimum number of tubercles elsewhere, pyrexia is comparatively slight until towards the end. Whether this explanation is the right one or not, it is certain that the absence of pyrexia even for a fortnight or more continuously by no means excludes tuberculous meningitis.

From the fortieth day onwards, the patient's condition grew very gradually but steadily worse. Cheyne-Stokes' respiration set in, with coma, and the infant died on the fifty-fourth day, the highest temperature, 104°F., being recorded just before death. This long duration of the illness from the time of appearance of the first acute symptoms is another point worthy of note, because it is often taught that an acute meningitis case which survives the fourth week is probably not tuberculous, but posterior basal.

The post-mortem examination showed the typical appearances of tuberculous meningitis and general tuberculosis. The brain and meninges exhibited nothing to account for the unilateral character of the paralysis. Tubercles and lymph were apparently symmetrical in their distribution in the common sites at the base of the brain; both lateral ventricles were greatly distended with turbid fluid, and the brain matter all round them was softened on both sides. There was no older caseous focus anywhere in the brain. There were moderate numbers of miliary tubercles in the lungs, liver and spleen; and very small numbers in the kidneys. There was a small caseous bronchial gland, which may have been the oldest tuberculous focus in the body; on the other hand, there were a dozen or more typical tuberculous ulcers in the intestine; it may well have been that the source of infection was there.

